# Understanding Barriers to Colon Cancer Screening Among Individuals Experiencing Housing Insecurity in Los Angeles

**DOI:** 10.7759/cureus.98313

**Published:** 2025-12-02

**Authors:** Arun Burra, Nikko Gonzales, Joshua Jiang, Shiliang Zhang, Mary Obasi, Ananya Eeraveni, Maria Garcia Jimenez, Sarah Goldgar

**Affiliations:** 1 Department of Medicine, University of California, Los Angeles, Los Angeles, USA; 2 College of Letters and Science, University of California, Los Angeles, Los Angeles, USA

**Keywords:** colon cancer screening, colorectal cancer screening, health disparity, homelessness, housing insecurity, primary care, unhoused

## Abstract

Background: Cancer is a leading cause of death among unhoused people, and the limited prior studies on colorectal cancer (CRC) screening in this group have shown low rates of screening compared to the general population.

Methods: Physicians administered surveys to patients at the University of California, Los Angeles Mobile Clinic who were experiencing homelessness or housing insecurity to determine rates of prior CRC screening, establish a baseline understanding of CRC screening, and elucidate barriers to screening. Physicians also administered a brief educational intervention about CRC screening and then administered a post-intervention survey to assess changes in participants’ understanding and preferences related to CRC screening.

Results: A total of 44 patients completed both the pre- and post-surveys and the educational intervention. Fewer than 50% of respondents aged 45 or older had a primary care provider (PCP) (n = 21) or had undergone CRC screening (n = 13). Patients who had health insurance - and particularly those who had a PCP - were more likely to have been offered and to have completed screening. CRC screening knowledge and self-efficacy increased after the brief educational intervention.

Conclusion: Helping unhoused patients obtain health insurance and establish care with a PCP may create opportunities to discuss preventive care and improve CRC screening rates.

## Introduction

People experiencing homelessness are more likely to suffer premature morbidity and mortality from all causes [1]. Cancer is a leading cause of death in this group [1,2]. Unfortunately, unhoused patients are both more likely to be at higher risk for colorectal cancer (CRC) and less likely to undergo CRC screening. One review found that in four studies in multiple states, fewer than 50% of unhoused patients were up to date on CRC screening compared to the national average of 67% [3]. When diagnosed with cancer, unhoused people have also been shown to experience worse clinical outcomes [4-6].

In the last decade, CRC incidence has risen among all adults younger than age 65 [7]. As a result, since 2021, the United States Preventive Services Task Force (USPSTF) has recommended starting CRC screening for average-risk individuals at age 45 rather than 50 [8-11]. As the median age of an unhoused person in California is 47 [12], most would be recommended to undergo CRC screening. Prior studies on CRC screening in unhoused people are limited; to our knowledge, none have been completed since the USPSTF expanded its screening recommendation.

Given the limited understanding around and significant need for cancer screening among the unhoused, the objectives of this study were to assess demographics at the clinic, evaluate CRC screening awareness, completion, and barriers, and assess the impact of an educational intervention on screening knowledge and self-efficacy among unhoused patients seen at the University of California, Los Angeles (UCLA) Mobile Clinic Project in Los Angeles. This is a street-side clinic staffed by medical students, medical residents, and attending physicians, and provides urgent care and social services to patients who are unhoused or facing housing insecurity.

## Materials and methods

Study setting

Internal medicine resident physicians administered surveys to patients at the UCLA Mobile Clinic Project. This is a street-side clinic that meets six times per month in the Hollywood neighborhood of Los Angeles, California. Surveys were collected during seven clinic sessions between October 2022 and February 2023 (see Appendix A for the surveys). The study was approved by the UCLA Institutional Review Board (IRB) (#22-000511), and the participants provided verbal consent. 

Pamphlet and survey development

A pamphlet with information on CRC, screening modalities, and local resources for accessing screening was developed using online resources from the United States Preventive Services Task Force (USPSTF) [13], American Gastroenterological Association [14], and American Cancer Society [15]. The pamphlet was assessed to be at a seventh-grade reading level (Flesch-Kincaid ease score of 70.7) and was reviewed by multiple practicing physicians for accuracy. Pre- and post-intervention surveys were developed to assess knowledge, attitudes, self-efficacy, and experiences around CRC screening, as well as demographic factors and perceived barriers to screening. The surveys consisted of multiple-choice questions and open-ended responses. Surveys and responses were administered in English or Spanish by resident physicians certified as bilingual translators. The English surveys and the pamphlet are available in the appendix (Appendices A and B).

Study procedures

Patients of the UCLA Mobile Clinic Project who were 18 years of age or older and had not previously participated were eligible for the study. Potential participants were approached while waiting for or after receiving services from the clinic and invited to participate. The pre-survey was conducted prior to the educational intervention. During the intervention, the pamphlet material was reviewed with patients by the internal medicine residents. The post-survey was given immediately after. A few patients chose to complete the survey on their own, but most preferred to have it read to them by a resident physician. Participants were paid $10 in grocery store gift cards after each survey completion ($20 total per participant for completing the pre- and post-survey). All data was de-identified and stored confidentially per IRB protocol.

Analysis

The results of the surveys were analyzed using relative risk ratios (when comparing rates of screening offers for patients over age 45 with primary care providers (PCPs) and those without PCPs, and for comparing patients who knew someone who had undergone CRC screening or had been diagnosed with CRC with patients who did not) and paired t-tests (when comparing overall test scores for patients pre- and post-intervention and comparing the proportions of patients who had indicated they “strongly agree” or “agree” with statements related to self-efficacy around CRC screening).

## Results

Patient demographics

A total of 44 participants participated during the study period. Nearly 90% (n = 39) of the participants met the criteria for homelessness as defined by the US Department of Housing and Urban Development, staying either in the street, a vehicle, a shelter, or temporary housing. The study population was racially diverse, reflective of the Los Angeles area and the unhoused population. The characteristics of the participants, including their reported race, gender, age, income, health insurance status, and whether they had a PCP, are noted in Table 1.

**Table 1:** Demographics of the study population

Survey respondents (n = 44)	n (%)
Gender	
Male	29 (66)
Female	15 (34)
Age	
≥45	30 (68)
<45	14 (32)
Race and ethnicity	
American Indian	2 (4)
Asian	6 (14)
Black or African American	10 (23)
Latino or Hispanic	8 (18)
White	10 (23)
Another race/ethnicity or did not disclose	8 (18)
Engagement with primary care	
Has a primary care provider (PCP)	21 (48)
Does not have a PCP	23 (52)
Last doctor’s appointment	
Within 1 year	29 (66)
Within 5 years	41 (93)
No prior visits	1 (2)
Housing status	
Permanent housing	5 (11)
Living in temporary housing, shelter, vehicle, or street	39 (89)
Employment	
Full- or part-time	6 (14)
Unemployed	36 (82)
Disabled	2 (4)
Income	
≥$400 per month	30 (68)
Less than $400 per month	12 (27)
Did not disclose	2 (4)
Insurance status	
Insured	32 (73)
Uninsured	12 (27)
Primary languages spoken	
Includes English	39 (89)
Does not include English	5 (11)
Education	
Completed high school or above	36 (82)
Some high school or less	8 (18)
Relationship status	
Married or in partnership	10 (23)
Single	34 (77)

Association of insurance coverage and primary care engagement with screening status

Most participants (73%, n = 32) reported having some form of health insurance (Table 1), primarily Medicaid, Medicare, or both (Table 2). Most patients had an appointment with a doctor within the prior five years, regardless of insurance status; however, less than half (48%, n = 21) had a PCP (Table 1).

**Table 2:** Insurance status and prior primary care visits

Insurance status	n	Has a PCP n (%)	Appt ≤ 1 y	Appt ≤ 5 y
Insured	32	18 (56%)	21	30
Medicare only	2	2 (100%)	0	2
Medicaid only	27	15 (55%)	18	25
Medicare and Medicaid	2	1 (50%)	2	2
Private	1	0	1	1
Uninsured	12	3 (25%)	8	11

Among insured patients who were at least 45 years old, nearly all (95%, n = 20) had heard of CRC screening, although only about half had been previously offered screening or previously completed some form of screening (Table 3). Among the uninsured patients over age 45, fewer (78%, n = 7) had heard of screening. An even smaller number (33%, n = 3) had been offered screening, and these same individuals all reported completing screening (Table 3).

**Table 3:** Insurance coverage and primary care physician (PCP) status compared with screening completion

	Screening status (age ≥ 45)		
	Heard of screening	Previously offered	Previously completed
Insurance status			
Insured (n = 21)	20 (95%)	11 (52%)	10 (48%)
Uninsured (n = 9)	7 (78%)	3 (33%)	3 (33%)
PCP			
Has PCP (n = 12)	12 (100%)	10 (83%)	9 (75%)
Does not have PCP (n = 18)	15 (83%)	4 (22%)	4 (22%)

As shown in Table 2, higher rates of insured patients (56%, n = 18) reported having a PCP, compared to only 25% (n = 3) of people in the uninsured group. As shown in Table 3, for the 12 patients over the age of 45 with PCPs, 83% had been previously offered screening compared with 22% of the 18 patients without PCPs (relative risk 2.63, 95% CI 1.11-6.21). Among all patients above age 45 who reported being offered screening (n = 14), at least 93% (n = 13) had completed screening. One respondent reported being offered screening, but did not disclose if it was completed. Seventy-five percent of patients with PCPs had completed screening (n = 9) compared to 22% of patients without a PCP (n = 4).

Effect of educational intervention on CRC screening knowledge and self-efficacy

**Figure 1:**
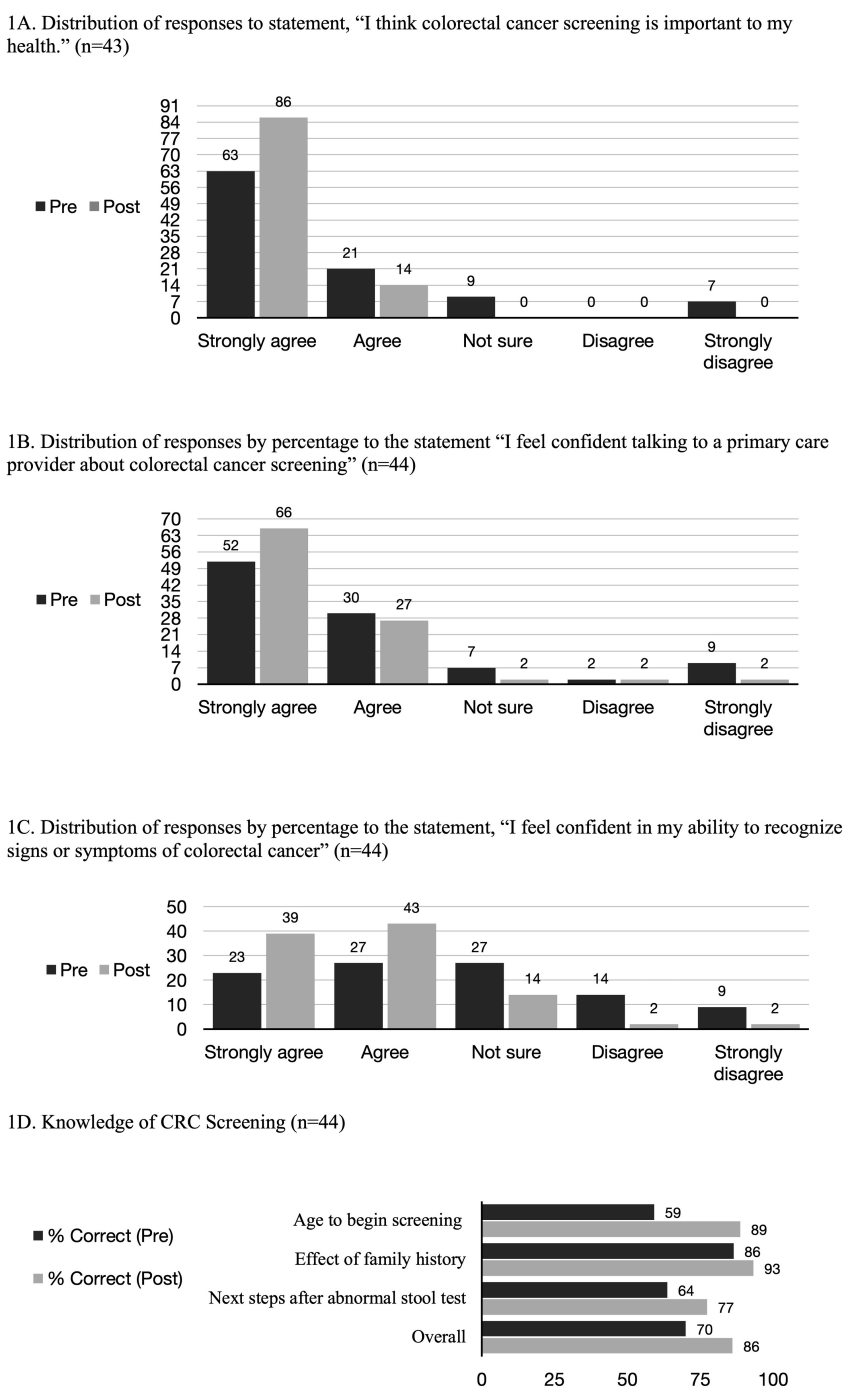
Pre- and post- educational intervention results

Patients were assessed before and after receiving a brief educational intervention using a pamphlet (Appendix B). Following this intervention, increased proportions of patients chose "strongly agree" or "agree" when asked to rate their agreement with questions around self-efficacy (Figures 1A, 1B, 1C). Specifically, more patients reported willingness to engage with a physician around CRC screening (p = 0.01), the ability to recognize symptoms of CRC (p = 0.001), the ability to articulate a preference for screening modality (i.e., stool testing, colonoscopy, or CT colonography), and agreed that screening was important (p = 0.002). 

**Figure 2:**
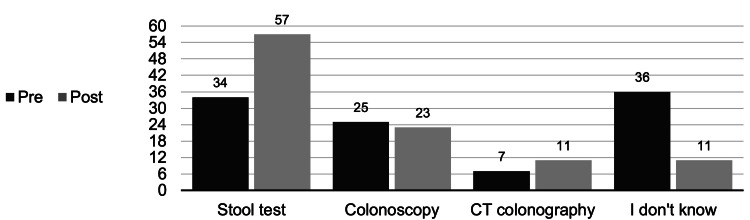
Pre- and post-intervention choice of colorectal cancer (CRC) screening modality by percentage (n = 44)

Patients were asked to identify the appropriate age to begin CRC screening for average-risk individuals, the effect of family history of CRC on screening age, and the recommendation for follow-up after an abnormal stool test. The average overall test scores improved significantly after the teaching intervention (70% to 86%, p = 0.00002) (Figure 1D). After this intervention, most patients indicated a preference for stool testing (Figure 2).

Self-identified barriers to CRC screening

The participants identified several barriers to CRC screening (Table 4). These included a lack of a PCP, concerns about the invasiveness of the colonoscopy, the expense of colon cancer screening, lack of transportation, lack of reliable access to a bathroom, concerns about belongings while going to the clinic or a procedure, fear around abnormal findings, and lack of health insurance. Only 7% (n = 3) felt that CRC screening was not worth the trouble (Table 4).

**Table 4:** Self-identified barriers to screening (n = 44)

Barriers	n (%)
I don’t have a primary care doctor.	17 (39)
I am worried about the colonoscopy being too invasive.	16 (36)
Colorectal cancer screening is expensive, and I don’t have the money right now.	16 (36)
I have no transportation to clinics.	15 (34)
I don’t have a reliable place with access to a bathroom to prep for a colonoscopy.	15 (34)
I don’t have anyone to watch my belongings while I am in the clinic or if I get a colonoscopy.	15 (34)
I am worried that they will find something wrong.	15 (34)
I don’t have health insurance.	15 (34)
I have other health issues that are more important right now.	11 (25)
I don’t have access to a bathroom to collect my own stool.	12 (27)
I don’t want to touch my own stool.	10 (23)
I don’t want to go through the process of prepping for a colonoscopy.	10 (23)
I’m not sure what to do for colorectal cancer screening.	9 (21)
I don’t think colorectal cancer screening is worth the trouble.	3 (7)

Personal knowledge of someone who was screened or diagnosed with CRC

Overall, patients’ knowledge of someone who had been screened for or diagnosed with CRC did not affect their own screening completion. For patients over age 45, patients with personal knowledge of someone who had completed CRC screening were more often screened compared to patients who did not (relative risk (RR) 1.33, 95% confidence interval (CI) 0.4-2.6). Similarly, patients with a personal knowledge of someone who had been diagnosed with CRC were more often screened than those who did not (RR 1.23, 95% CI 0.4-2.4). These findings were not statistically significant.

## Discussion

Our study showed poor rates of completion of CRC screening among the unhoused. Consistent with prior literature [16-18], fewer than 50% of patients over age 45 had completed CRC screening, and a majority reported never being offered it in the first place. The racial and gender demographics of the surveyed group in our study were diverse, and similar to those documented in the Los Angeles Homeless Services Authority Point-in-Time Count in 2024 [19] - most patients identified as male and as non-White [20,21]. Notably, the majority of surveyed patients had health insurance (a finding that would not necessarily be replicated in states that did not expand Medicaid eligibility under the Affordable Care Act), a source of income, and had seen a physician within the prior year. Even still, most patients did not have an established PCP, which is similar to previously published data. Indeed, a cross-sectional study of unhoused people in Canada, a country with universal health insurance, showed that a minority had a PCP [22].

Various studies demonstrate that limited health literacy (defined by the Institute of Medicine as “the capacity to obtain, process, and understand basic health information and services to make appropriate decisions”) is also associated with lower rates of CRC screening, as well as increased overall mortality from all causes; one study also showed surveys can help inform patient’s choice of screening and motivation to complete screening [23,24]. Our brief educational intervention showed significant improvements in relevant medical knowledge as well as self-efficacy, although we were unable to assess for effects on long-term knowledge or behavioral changes around CRC screening. Still, increased CRC screening education and counseling, emphasizing the importance of screening and addressing misconceptions around it, is valuable. However, addressing health education - while important - is insufficient to address CRC screening and other important health disparities among people experiencing homelessness, given the unique structural challenges that unhoused patients face. Systems-level and policy interventions are needed to ensure adequate CRC screening, as evidenced by the improved CRC screening rates observed among patients who had health insurance in our study.

Interestingly, patients who were eligible for screening and had a PCP had an even greater magnitude of improvement in CRC screening completion rates (compared to those who did not have a PCP) than similarly aged patients with health insurance (compared to those who did not have health insurance). PCPs can provide valuable education as well as special attention to preventive screening. Not having a PCP was also the most cited barrier to CRC screening among the patients we surveyed. This supports the significance of establishing care with a PCP as a public health intervention, as well as the significant progress the medical establishment has yet to make in engaging with the unhoused community. In addition, the significant percentage of patients with PCPs who reported never being offered screening also highlights an important care gap, given that cancer is a leading cause of mortality in the unhoused community and that CRC may be prevented through appropriate screening.

Prior studies have elucidated various barriers unhoused patients face in completing CRC screening. This includes difficulties with access to physicians and navigating the healthcare system, low rates of provider recommendation, mistrust in the medical system (sometimes compounded by a history of medical trauma), and a lack of private space for testing or bowel preparation [25,28]. In our study, which reproduced these findings, the most significant barriers reported were financial and logistical (e.g., having a reliable bathroom and transportation to a clinic). Of note, despite the profound subsistence needs inherent to homelessness, a small minority of respondents indicated that CRC screening was “not worth the trouble,” and a minority reported having medical conditions they thought were more important than preventive screening. In light of these barriers, prior studies [25,26] have recommended stool testing as first-line for primary prevention, providing private spaces for obtaining a sample, and funding patient navigators (who can assist with scheduling appointments and transportation). Patient navigators have been shown to improve CRC screening rates among unhoused and low-income patients [27,28]. We also agree with prioritizing the use of stool-based testing modalities, which reduce barriers to screening, are more cost-effective, and were preferred by the patients in our study.

While stool testing is much easier for both patients and healthcare systems to arrange, healthcare systems must also be prepared to set up follow-up colonoscopy and support patients who have abnormal stool tests. This presents unique challenges; for example, one study showed that incentives, patient navigators, and reminders can improve stool test return rates, but colonoscopy follow-up did not improve significantly [27]. While another study did show that for black and non-English speaking patients, using patient navigators increased CRC screening rates, colonoscopy, and adenoma detection [29], so may still be an important way to facilitate initial testing and follow-up colonoscopy. Patients in our study cited concerns about the invasiveness of colonoscopy and preferred to start with stool testing. Bowel preparation before colonoscopy requires having frequent loose bowel movements, and poor preparation can preclude diagnosis or necessitate repeat colonoscopy. Schwartz et. al offer colonoscopy-specific medical respite programs or hotel vouchers as a possible solution, given the logistical and financial challenges of admitting patients to the hospital for screening colonoscopy and the regularity with which medical respite programs do not have bathroom capacity for patients who need to prepare for colonoscopy [25]. 

Based on our findings, there are several primary strategies to improve CRC screening in those facing housing insecurity, including offering stool-based testing, tying patients to PCPs, and ensuring patients have health insurance. Other areas include using patient navigators, partnering with street medicine teams or shelters to offer screening, not delaying preventative care even when patients have many other needs, offering incentives to encourage screening completion [27], and recognizing how stable housing and policy changes around access to housing and healthcare make these types of preventative services more feasible. While many of these methods have been proposed or tested in lower-income populations as noted above, further study is needed to determine which interventions are most effective for improving CRC screening in the unhoused.

Limitations of our study include the recall bias inherent to survey responses, limited sample size, and sampling bias possibly introduced in surveying UCLA Mobile Clinic patients. Although many in our sample are not well-connected to health care, by surveying at a street-side clinic, it is possible that these individuals are better connected or more receptive to medical services than the average unhoused person, as they had chosen to seek medical care on that day. Furthermore, there are significant differences in health insurance policies and medical resources available to unhoused people nationwide, so this sample taken at a single location in Los Angeles may vary from the average unhoused American. We will note that 11% of our study population reported being housed, although they were still accessing services at a street-side clinic. In our experience, these are often patients who were previously unhoused and developed a trusting relationship with our street medicine providers during that time, so they continue to access services, particularly in the early transition to being housed. They remain a vulnerable group with ongoing barriers to health.

Limitations of our data collection include potential social desirability bias introduced through resident physician-led data collection, though all participants were allowed to give written, anonymous responses, and neutral phrasing was used in this process. We also did not use a validated measure for our survey and were unable to measure durable improvements in knowledge, given that patients were surveyed only once immediately after the educational intervention, thus capturing short-term recall rather than durable learning. A future study should consider ways to follow patients longitudinally to assess long-term retention and behavioral changes to better understand how education and increased self-efficacy affect this. We also did not assess patients in languages other than English and Spanish. Given that non-English speakers have been shown to have poorer health literacy [30] and health outcomes in the primary care setting [31], this is an important community to study as well as a likely source of sampling bias.

Despite the limitations, our study is the first to assess demographics and barriers to CRC screening in the unhoused community in the Los Angeles area in decades, the first to assess CRC screening since the updated USPSTF guidelines, and the first to elicit unhoused patient preferences around CRC screening modality. Further studies need to assess a larger number of patients in both urban and rural areas and assess interventions to improve rates of both CRC screening and follow-up colonoscopies after abnormal results.

## Conclusions

Our study showed a minority of unhoused patients undergo CRC screening. It also showed that brief educational interventions are associated with improved CRC screening knowledge and self-efficacy and that health insurance enrollment, offering patients CRC screening, and most notably having a PCP is associated with improved screening rates. Street medicine providers should prioritize helping patients obtain health insurance, establish care with a PCP, and maximize opportunities to discuss preventive care during each encounter.

## Figures and Tables

**Figure 3:**
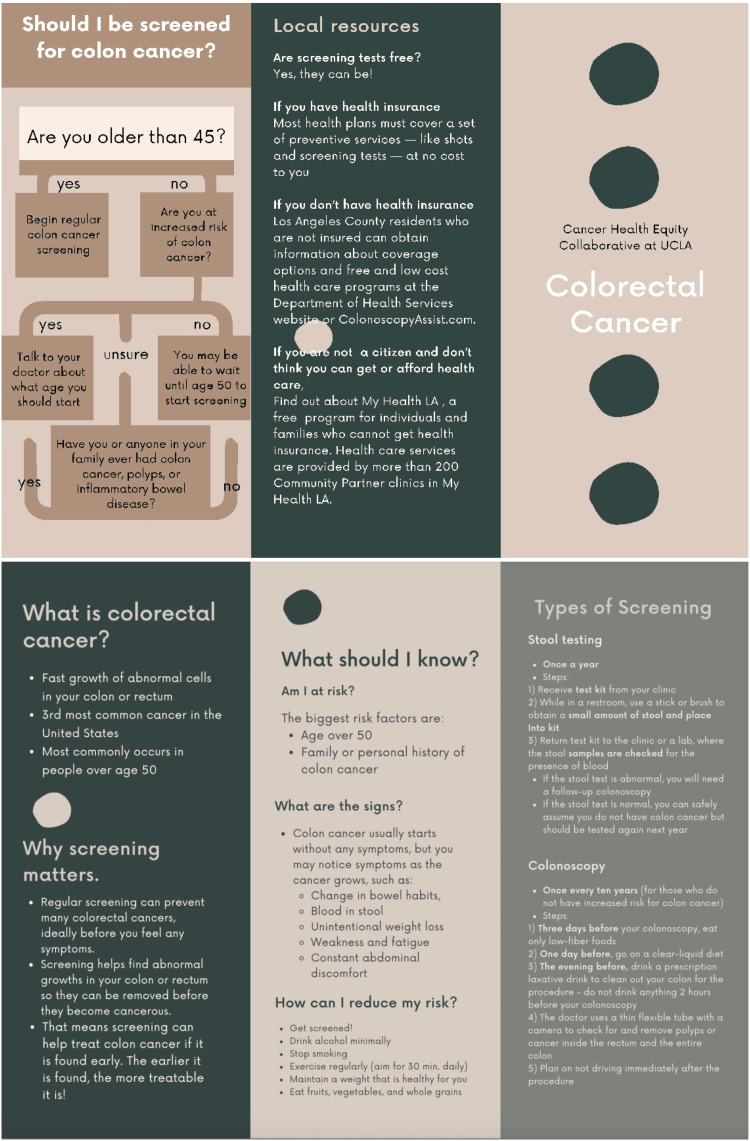
Colorectal cancer information pamphlet

## Appendices

Appendix A: pre- and post-surveys

Colorectal Cancer Screening Pre-Survey

We are contacting you on behalf of UCLA Health because we are working on a research study about colorectal cancer screening.  We would like to ask you some questions about your medical history and your thoughts about colorectal cancer. This survey is voluntary, and your decision to participate will have no effect on the care you receive or on any relationship with your health care team.

The study involves a 5-minute survey (below) that asks you general questions about your history of prior colorectal cancer screening. There are minimal risks to you, but great benefits to our understanding of colorectal screening in patients like you.  If you complete the survey, we will give you a $15 gift card to thank you for your time and participation.

First Name: ___________________

Last Name: ___________________

Date of Birth: __________________

Gender: ______________________

Previous Medical and Colorectal Cancer Screening History

Do you have a primary care doctor?

a.          Yes

b.          No

When was the last time you had an appointment with a doctor?

a.          Within a year

b.          Within 5 years

c.          Within 10 years

d.          Never

What are the different types of colorectal cancer screening you have heard of before? (Circle all that apply)

a.          Stool test (a sample of your stool was taken and checked for blood)

b.          Flexible sigmoidoscopy (a test where your doctor looks at the last part of your bowels with a camera scope)

c.          Colonoscopy (a test where your doctor looks at your entire large bowel with a camera scope)

d.          CT colonography (through taking pictures with a CAT scan)

e.          None of the above

What different types of colorectal cancer screening have you been offered before? (Circle all that apply)

a.          Stool test (a sample of your stool was taken and checked for blood)

b.          Flexible sigmoidoscopy (a test where your doctor looks at the last part of your bowels with a camera scope)

c.          Colonoscopy (a test where your doctor looks at your entire large bowel with a camera scope)

d.          CT colonography (through taking pictures with a CAT scan)

e.          None of the above

What different types of colorectal cancer screening have you had before? (Circle all that apply)

a.          Stool test (a sample of your stool was taken and checked for blood)

b.          Flexible sigmoidoscopy (a test where your doctor looks at the last part of your bowels with a camera scope)

c.          Colonoscopy (a test where your doctor looks at your entire large bowel with a camera scope)

d.          CT colonography (through taking pictures with a CAT scan)

e.          Other screening test: ______________________

f.          I have never been screened for colorectal cancer before

g.         I do not remember

What challenges have you had with colorectal cancer screening?

____________________________________________________________________________________

____________________________________________________________________________________

Do you know someone who has had colorectal cancer screening?

a.          Yes

b.          No

c.          Not sure

Do you know someone who has had colon or rectal cancer?

a.            Yes

b.           No

c.            Not sure

Understanding Colorectal Cancer

Please indicate to what extent you agree or disagree with the following statement:

I think colorectal cancer screening is important for my health.

a.          Strongly agree

b.          Agree

c.          Not sure

d.          Disagree

e.          Strongly disagree

Please indicate to what extent you agree or disagree with the following statement:

I feel confident talking to a primary care provider about colorectal cancer screening

a.          Strongly agree

b.          Agree

c.          Not sure

d.          Disagree

e.          Strongly disagree

Please indicate to what extent you agree or disagree with the following statement:

I feel confident in my ability to recognize signs or symptoms of colorectal cancer.

a.          Strongly agree

b.          Agree

c.          Not sure

d.          Disagree

e.          Strongly disagree

At what age should average-risk people be screened for colorectal cancer?

a.          At age 45 and above

b.          At age 50 and above

c.          At age 65 and above

d.          I don’t know

Can a family history of colorectal cancer affect whether you should be screened earlier?

a.          Yes

b.          No

c.          I don’t know

What do you have to do if your stool test is abnormal?

a.            Take the test again in 6 months

b.           Take the test again in 1 year

c.            Improve your diet by eating more vegetables and fruits

d.           Get a colonoscopy to further evaluate

If you were to get colorectal cancer screening now, what type of screening would you pursue?

a.          Stool test (a sample of your stool was taken and checked for blood)

b.          Flexible sigmoidoscopy (a test where your doctor looks at the last part of your bowels with a camera scope)

c.          Colonoscopy (a test where your doctor looks at your entire large bowel with a camera scope)

d.          CT colonography (through taking pictures with a CAT scan)

Do you know where to go if you want to get colorectal cancer screening? Y/N

If you answered yes, please indicate location: ________________________

Demographics

Race and ethnicity (please circle any that apply)

a.          American Indian

b.          Asian

c.          Black or African American

d.          Hispanic or Latinx

e.          Native Hawaiian/Pacific Islander

f.          White

Employment:

a.          Part-time

b.         Full-time

c.          Unemployed (Seeking___, Not seeking___)

d.         Disabled

e.          Retired

Health Coverage:

a.           MediCal (Medicaid)

b.          Medicare

c.           VA

d.         My Health LA

e.          Private

f.          None

g.         Other _____________

Personal Income:

a.          >$400 a month

b.          <$400 a month

Housing status:

a.           Permanent Housing

b.          Temporary Housing

c.           Shelter

d.         Vehicle

e.          Street

What is your primary language:

a.          English

b.          Spanish

c.          Chinese

d.          French

e.          Tagalog

f.          Korean

g.          Vietnamese

h.          Farsi

i.           Arabic

j.           Other (please specify): ________________

Highest level of education:

a.          Some primary/middle school

b.          Some high school

c.          Completed high school/GED

d.          Some college

e.          Completed college degree

Married or in partnership:

a.          Yes

b.          No

Colorectal Cancer Screening Post-Survey

We are contacting you on behalf of UCLA Health because we are working on a research study about colorectal cancer screening.  We would like to ask you some questions about your medical history and your thoughts about colorectal cancer. This survey is voluntary, and your decision to participate will have no effect on the care you receive or on any relationship with your health care team.

The study involves a 5-minute survey (below) that asks you general questions about your history of prior colorectal cancer screening. There are minimal risks to you, but great benefit to our understanding of colorectal screening in patients like you.  If you complete the survey, we will give you a $15 gift card to thank you for your time and participation.

First Name: ___________________

Last Name: ___________________

Understanding Colorectal Cancer

Please indicate to what extent you agree or disagree with the following statement:

I think colorectal cancer screening is important to my health.

a.          Strongly agree

b.          Agree

c.          Not sure

d.          Disagree

e.          Strongly disagree

Please indicate to what extent you agree or disagree with the following statement:

I feel confident talking to a primary care provider about colorectal cancer screening

a.          Strongly agree

b.          Agree

c.          Not sure

d.          Disagree

e.          Strongly disagree

Please indicate to what extent you agree or disagree with the following statement:

I feel confident in my ability to recognize signs or symptoms of colorectal cancer.

a.          Strongly agree

b.          Agree

c.          Not sure

d.          Disagree

e.          Strongly disagree

At what age should average-risk people be screened for colorectal cancer?

a.          At age 45 and above

b.          At age 50 and above

c.          At age 65 and above

d.          I don’t know

Can a family history of colorectal cancer affect whether you should be screened earlier?

a.          Yes

b.          No

c.          I don’t know

What do you have to do if your stool test is abnormal?

a.            Take the test again in 6 months

b.           Take the test again in 1 year

c.            Improve your diet by eating more vegetables and fruits

d.           Get a colonoscopy to further evaluate

If you were to get colorectal cancer screening now, what type of screening would you pursue?

a.          Stool test (a sample of your stool was taken and checked for blood)

b.          Flexible sigmoidoscopy (a test where your doctor looks at the last part of your bowels with a camera scope)

c.          Colonoscopy (a test where your doctor looks at your entire large bowel with a camera scope)

d.          CT colonography (through taking pictures with a CAT scan)

Do you know where to go if you want to get colorectal cancer screening? Y/N

If you answered yes, please indicate location: ________________________

Self-Perceived Barriers to Colorectal Cancer Screening

Please put a check mark in the line next to any obstacle that you feel may prevent you from getting colorectal cancer screening.

Afterwards, please review your checked boxes and circle the three obstacles you feel are most difficult for you to overcome.

____ I don’t have a primary care doctor.

____ I don’t have health insurance.

____ Colorectal cancer screening is expensive, and I don’t have the funds right now.

____ I don’t think colorectal cancer screening is worth the trouble.

____ I have other health issues that are more important right now.

____ I’m not sure what to do for colorectal cancer screening.

____ I don’t want to go through taking a sample of my own stool.

____ I don’t want to go through prepping for a colonoscopy.

____ I am worried about the colonoscopy being too invasive.

____ I don’t have a reliable place with access to a bathroom in order to prep for a colonoscopy.

____ I don’t have anyone to watch my belongings while I am in clinic or if I get a colonoscopy.

____ I have no transportation to clinics.

____ I am worried that they will find something wrong.

Appendix B: pamphlet
